# The anorexic effect of DL-fenfluramine is dependent on animals' habituation to different food types

**DOI:** 10.3389/fnint.2022.1010181

**Published:** 2022-11-16

**Authors:** Sun Shin Yi, SuJean Choi

**Affiliations:** ^1^Department of Biomedical Laboratory Science, College of Medical Sciences, Soonchunhyang University, Asan, South Korea; ^2^BK21 Four Program, Department of Medical Sciences, Soonchunhyang University, Asan, South Korea; ^3^Department of Biomedical Sciences, College of Health Sciences, Marquette University, Milwaukee, WI, United States

**Keywords:** DL-fenfluramine (FEN), tolerance, normal chow diet (NC), western diet (WD), habituated food

## Abstract

**Background:**

As rates of obesity and diabetes have increased dramatically over the past few decades, the use of anti-obesity drugs has now become a routine therapeutic measure. However, the pharmacological effects of chronic use of these drugs in humans frequently lead to reduced efficacy in reducing appetite and body weight through as-yet-unidentified mechanisms. An example of this can be found in animal studies where the appetite suppressant DL-fenfluramine (FEN) is chronically administered and its tolerance develops in animals and humans. The appetite effects of FEN are typically measured in several animal studies by the feeding changes in a balanced standard diet. To determine whether FEN differentially altered appetite suppression in animals with long-term expression with different macronutrient diet compositions, its anorexic effects were measured specifically in male rats that had previously been chronically maintained on normal chow (NC) or a high-fat and high-carbohydrate western diet (WD).

**Methods:**

Three experiments were conducted by feeding the animals either NC or WD for 1 month to habituate them with their diet. Animals maintained on either NC or WD were subsequently offered both diet options *ad libitum* for a 2- or 7-day adaptation period while receiving daily systemic FEN treatment.

**Results:**

The results suggested that long-term habituated food affected the food preference of animals and their appetite even after chronic systemic FEN administration. Therefore, the effectiveness and success or failure of repeated use of chronic anti-obesity drugs may depend on habituated food type.

**Conclusion:**

The appetite suppressant effect was found to be determined by the palatability of a specific macronutrient and the habituated food rather than by a change in the concentration of the administered FEN. This results in a critical analysis of the rationale for taking medication considering the patient's past dietary habits to achieve successful weight loss.

## Introduction

Due to the global issues related to overweight and obesity, increasing attention has been paid to the regulatory mechanisms of pharmacological agents used to treat obesity. Although pharmacological agents such as DL-fenfluramine (FEN) effectively reduce body weight and fat deposits substantially (Boisvert et al., 2011), adverse effects from these anorectic and body weight-reducing agents are common, and rebound weight gain can occur after drug withdrawal (Choi et al., 2002; Fernstrom and Choi, 2008). Moreover, both human and experimental animals chronically treated with FEN have demonstrated that anorectic and weight-reducing effects diminish over time (Choi et al., 2002; Fernstrom and Choi, 2008). However, what were infrequently considered are the preferences in macronutrient composition found in individuals' eating habits, which can influence lifelong habitual eating patterns (French et al., 1994; Lupton, 2000; Lartey et al., 2018). Individuals with obesity stemming from poor eating habits and a strong desire for high caloric density foods may require medically supervised treatment (Adler and Stewart, 2009; Li and Cheung, 2011; Poulton et al., 2016). Yet, clinical tools to suppress appetite and promote weight loss typically involve serotonin and/or norepinephrine modulators with varying degrees of success (Bello and Liang, 2011; Li and Cheung, 2011; Gill et al., 2020). Variability in individual weight loss may be triggered by signaling factors such as synaptic concentrations of catecholamines and food habits, that can arise from chronic macronutrient intake patterns (French et al., 2001; Adler and Stewart, 2009; Halford et al., 2010; Kearney, 2010; Li and Cheung, 2011; Poulton et al., 2016; Gopal et al., 2020). Few studies investigating serotonin-mediated appetite suppression have examined past macronutrient intake for the predictive value of appetite suppression.

In this study, we conducted several experiments in which rats were habituated to either the normal chow (NC) or western diet (WD) for 1 month and then given *ad libitum* access to both foods in conjunction with chronic FEN administration to determine whether the effects of long-term macronutrient history affected the efficacy of FEN in suppressing feeding behavior. Specifically, we examined whether repeated systemic administration of a serotonin-promoting drug, FEN, would result in differences in the magnitude of anorexic actions in animals with a history of high-fat and high-carbohydrate food (WD) consumption relative to those maintained on NC.

## Materials and methods

### Animals and diets

**Figure 1:**
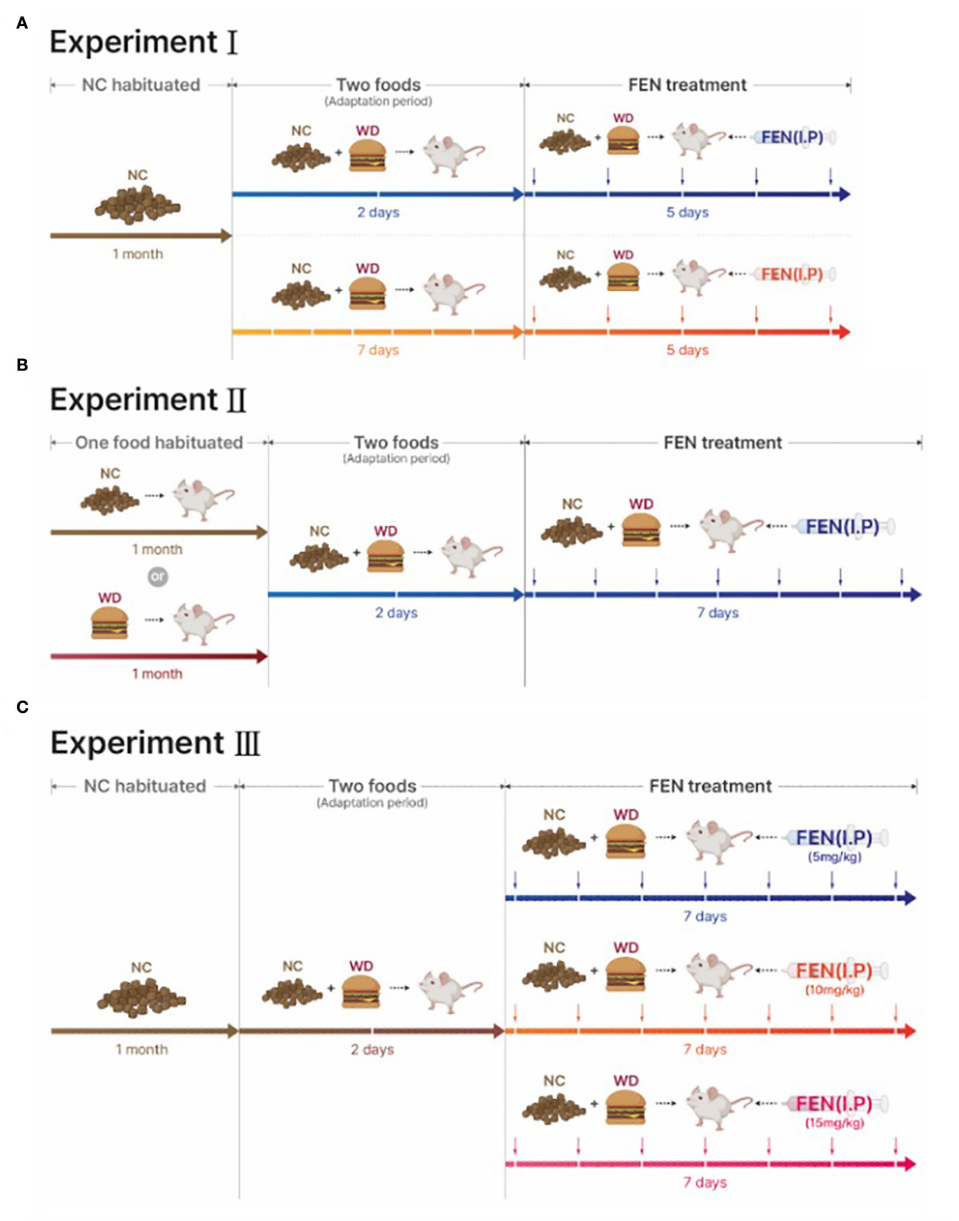
The timetables of three experiments are designed to discover the effects of eating behavior on the self-selecting adaptation time for normal chow (NC) and palatable western diet (WD) foods by fenfluramine (FEN) treatment. **(A)** NC-habituated animals were acclimatized to the two types of food for 2 or 7 days and then administered FEN. **(B)** NC- or WD-habituated animals were treated with FEN after 2 days of adaptation to both diets. **(C)** Three different concentrations (5, 10, and 15 mg/kg/day) of FEN were administered to NC-habituated animals after a 2-day acclimatization period to the two diets (NC and WD). NC, normal chow, WD, western diet.

Adult male Sprague–Dawley rats (weight, 225–250 g; Harlan, Madison, WI, USA) were housed individually in a climate-controlled room with a 12:12 h light-dark cycle (lights on at 05:00 h). Animals had free access to NC (Harlan 8604 formulation) or WD (Rodent Chow #D12079B, Research Diet, Inc., New Brunswick, NJ, USA), and water unless otherwise stated. The standard NC provides 3.0 kcal/g of energy and WD provides 4.7 kcal/g of energy; specific diet composition is shown in Table 1. After 1 month of habituation to either diet, animals were given access to both NC and WD during an adaptation period of either 2 or 7 days (see Figure 1). After this adaption period, each diet group was further divided into two treatment groups (saline or FEN).

**Table 1:** Comparison of food ingredients ratio between NC and WD in this study.

**Diets**	**Calories from carbohydrate**	**Calories from protein**	**Calories from fat**
Harlan normal chow (3.0 kcal/g)	54% (↑11% Higher)	32% (↑15% Higher)	14%
Research diet western diet (4.7 kcal/g)	43%	17%	41% (↑27% Higher)

Fenfluramine concentrations (Sigma-Aldrich, St. Louis, MO. USA) of 5, 10, or 15 mg/kg/day were administered intraperitoneally (I.P.) 2 h before lights were turned off. Body weight and food intake were measured daily, and grams consumed were expressed in kilocalories (kcal/day). There are a few records of using FEN at 5 or 10 mg/kg in the study by Choi et al. (2002, 2006, 2011). This is the rationale for our dosing concentration. Except for Experiment III, the concentration of FEN used in all other experiments was 5 mg/kg/day. All procedures were performed in accordance with the guidelines and approval of the Marquette University Institutional Animal Care and Use Committee (IACUC).

### Experiment I: Changes in the efficacy of systemic FEN in animals given a choice between NC or WD food after chronic NC experience

Animals habituated to NC for 1 month were allowed to choose between NC or WD for 2 or 7 days. Subsequently, we administered FEN (5 mg/kg/day) or vehicle (saline) daily I.P. for 5 days after the 2- or 7-day adaption period. We measured food intake for 24 h, which is presented as kilocalories per day (Figure 1A).

### Experiment II: Changes in the efficacy of systemic FEN and diet choice after chronic NC or WD experience

Animals previously habituated to NC or WD for 1 month were allowed to choose between NC and WD for 2 days followed by 7-day administration of vehicle or FEN (NC-SAL, NC-FEN, WD-SAL, and WD-FEN). After vehicle or FEN (5 mg/kg/day; I.P.) administration, animal dietary intake was measured every 24 h (Figure 1B).

### Experiment III: Observing the effects of different FEN concentrations on the food preferences of NC-habituated animals

Experiment III proposes to examine whether changes in the concentration of FEN affect the food preferences of NC-habituated animals. Therefore, we habituated animals to NC food for 1 month and then separated them into four groups, including a vehicle (SAL), FEN (5, 10, and 15 mg/kg/day). NC-habituated animals were given 2 days to adapt to both foods (NC and WD); vehicle and three different concentrations of FEN were then administered daily for 7 days (Figure 1C). Intake volumes were measured and converted to total daily kilocalorie intake.

### Data analysis

Data are presented as means ± standard errors of the mean (SEM). All statistical analyses were performed using a two-tailed Student's *t*-test for a comparison of two groups or analysis of variance (two-way ANOVA) followed by Dunnett's *post*-*hoc* test using the statistical software package (GraphPad Prism version 9.0, GraphPad Software, Inc., San Diego, CA, USA) for a comparison of multiple groups. Statistical significance was considered when *p* < 0.05.

## Results

### Experiment I: Varying periods of dietary self-selection alter FEN-induced appetite suppression for specific diet compositions

**Figure 2:**
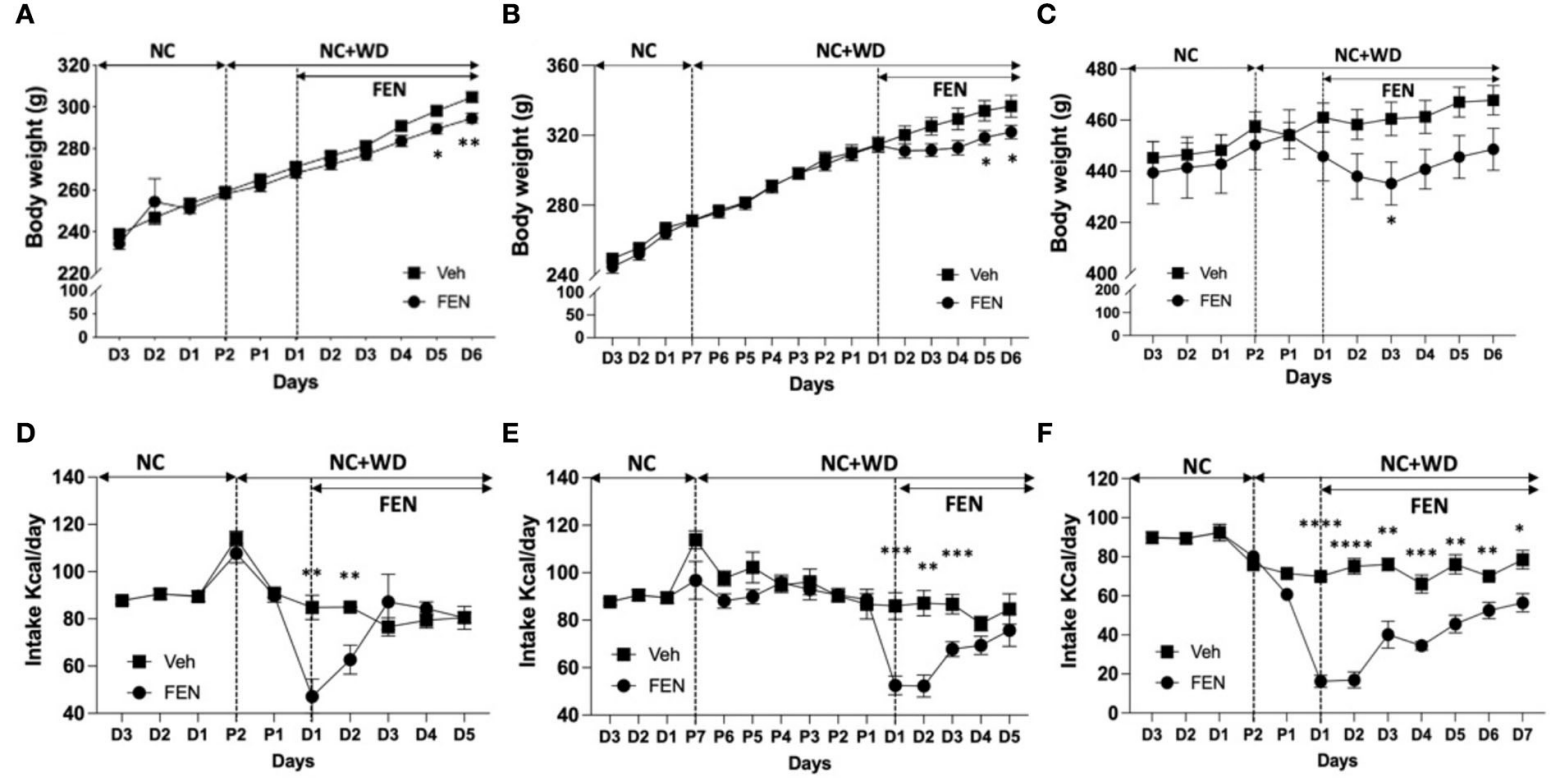
Weight change by FEN according to the NC+WD exposure period of NC- or WD-adapted animals. **(A,B)** shows the weight change of animals caused by FEN when two kinds of food (NC + WD) were served simultaneously for 2 or 7 days for NC-adapted animals, respectively, and **(C)** shows the weight change in WD-adapted animals caused by FEN after the access of NC + WD free for 2 days. In the same way as above, after NC-adapted or WD-adapted animals were exposed to two types of NC and WD for 2 days **(D,F)** or 1 week **(E)**. Statistical significance was confirmed for both body weight and caloric intake results between veh- and FEN-treated by two-way ANOVA [**(A,B,D–F)**, *p* < 0.0001; C, *p* = 0.0033]. It mainly appears at the beginning of FEN administration. All animals were habituated to NC or WD for 1 month, and weight loss and low appetite were observed after FEN administration. Error bars were expressed as mean ± standard errors of the mean (SEM). **p < 0.01; *p < 0.05 vs. Veh.

**Figure 3:**
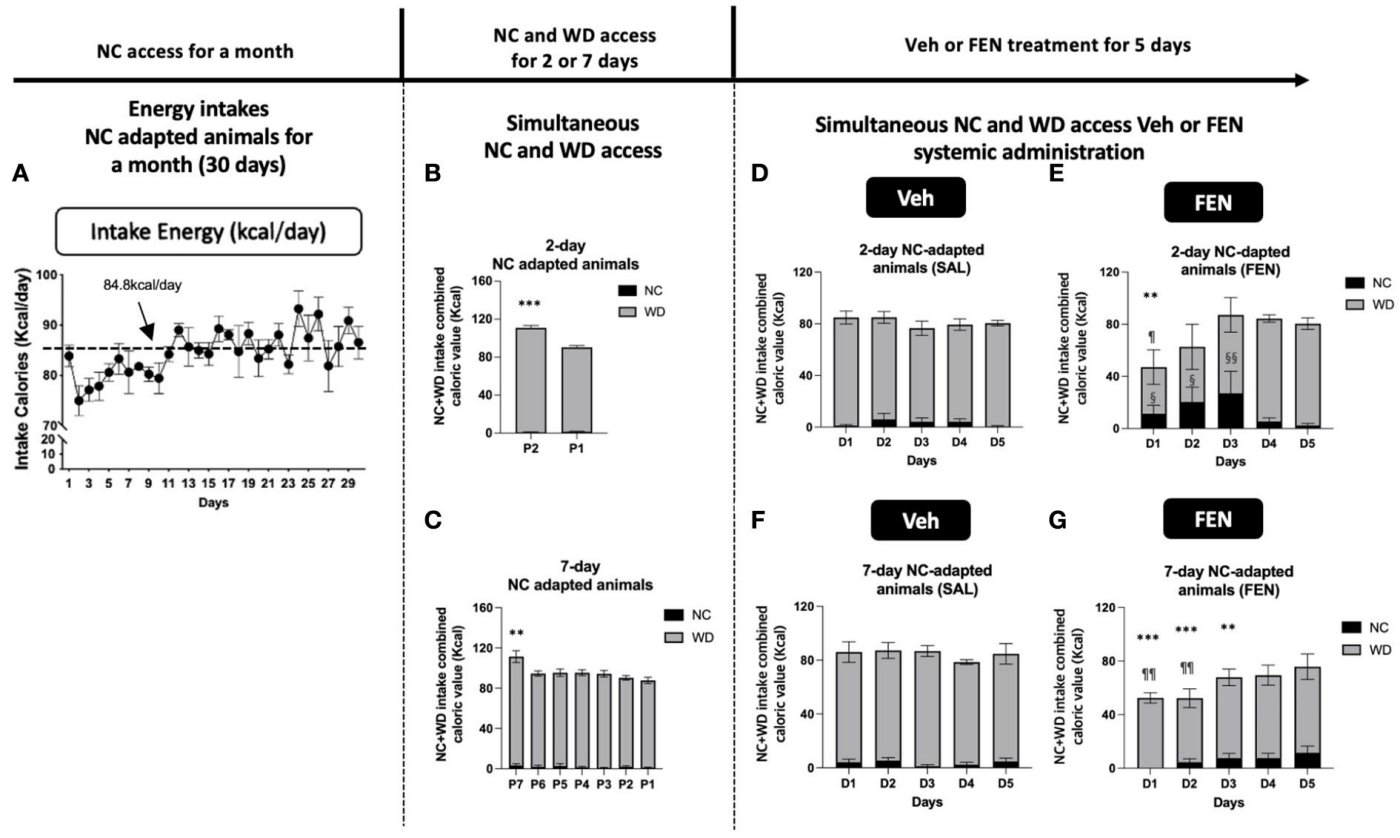
NC-habituated animals (1 month) are divided into two groups according to their adaptation periods [2 **(A)** and 7 days **(B)**]. The average daily food intake and energy intake was 28.3 g and 84.8 kcal, respectively. Animals treated with both NC and the novel food (WD) showed high total calorie intake on the 1st day and subsequently reduced their caloric intake **(B,C)**. Total caloric intake was drastically reduced on the 1st day after systemic FEN (5 mg/kg/day) administration in both groups, after which total caloric intake was gradually restored to the normal in both groups **(D–G)**. Error bars were expressed as mean ± SEM. ***p < 0.005; **p < 0.01; *p < 0.05 vs. Veh for each day; ^§§^*p* < 0.01; ^§^*p* < 0.05 vs. NC consumed by the Veh group for each day; ^¶¶^*p* < 0.01; ^¶^*p* < 0.05 vs. WD consumed by the Veh group for each day (Student's two-tailed *t*-test).

We observed changes in body weight (Figures 2A–C) and changes in the animals' food intake (Figures 2D–F) after FEN administration. When animals adapted to NC (Figures 2A,B) or WD (Figure 2C) were then freely accessed to the two types of food for 2 days and then posted FEN administrations, statistical significance was observed between the Veh- and FEN-treated groups by two-way ANOVA (Figures 2A,B, each *p* < 0.0001 and Figure 3C; *p* = 0.0033). Statistical significance of body weight by day after FEN administration was observed in Figures 2A,B, D5–6 and 2C, D3. In contrast, total caloric intake was observed to show a significant difference at the beginning of FEN administration, but gradually decreased over time.

Initially, both groups of animals show a marked increase in total caloric intake during the adaption period (2nd and 7th day), primarily from WD 17.2%/day (^***^p < 0.005), and 12.7%/day (^**^p < 0.01) increase compared to their normal consumption (84.8 kcal/day), respectively. As observed in our previous studies (Choi et al., 2006; Boisvert et al., 2011), total calories consumed decreased markedly after the 1st day of systemic FEN administration compared with that of saline treatment (57.3% decrease (^**^p < 0.01), 2 days self-selection; 39.7% decrease (^***^p < 0.005), 7 days self-selection; a statistically significant difference at D1) (Figures 3E,G), but then gradually returned to preinjection levels by day 5 (D5; SAL vs. FEN, *p* = 0.989; Figure 3E, and *p* = 0.366; Figure 3G). Interestingly, animals that were allowed only 2 days to self-select from both NC and WD showed a notable suppression in calories of WD (^¶^*p* < 0.05) at D1 and a concomitant significant increase in calories obtained from NC for up to 3 days during systemic FEN treatment (^§§^*p* < 0.01; ^§^*p* < 0.05; Figure 3E). In contrast, animals allowed for 7 days to self-select both NC and WD also showed a notable suppression in total calories (^***^p < 0.005; ^**^p < 0.01; Figure 3G), but demonstrated little interest toward NC as chronic FEN treatment showed no increase in NC consumption. On the other hand, it was confirmed that the consumption of WD in the FEN-administered group was significantly reduced compared to that of the Veh-administered group in the first 2 days (^¶¶^*p* < 0.01; Figure 3G). When both types of calorically valued foods could be accessed simultaneously, a 2-day duration was sufficient to prejudice a preference toward palatable food during FEN administration.

### Experiment II: Differences in food preferences after FEN systemic administration with NC- or WD-habituated animals after the 2-day food-adaptation period

**Figure 4:**
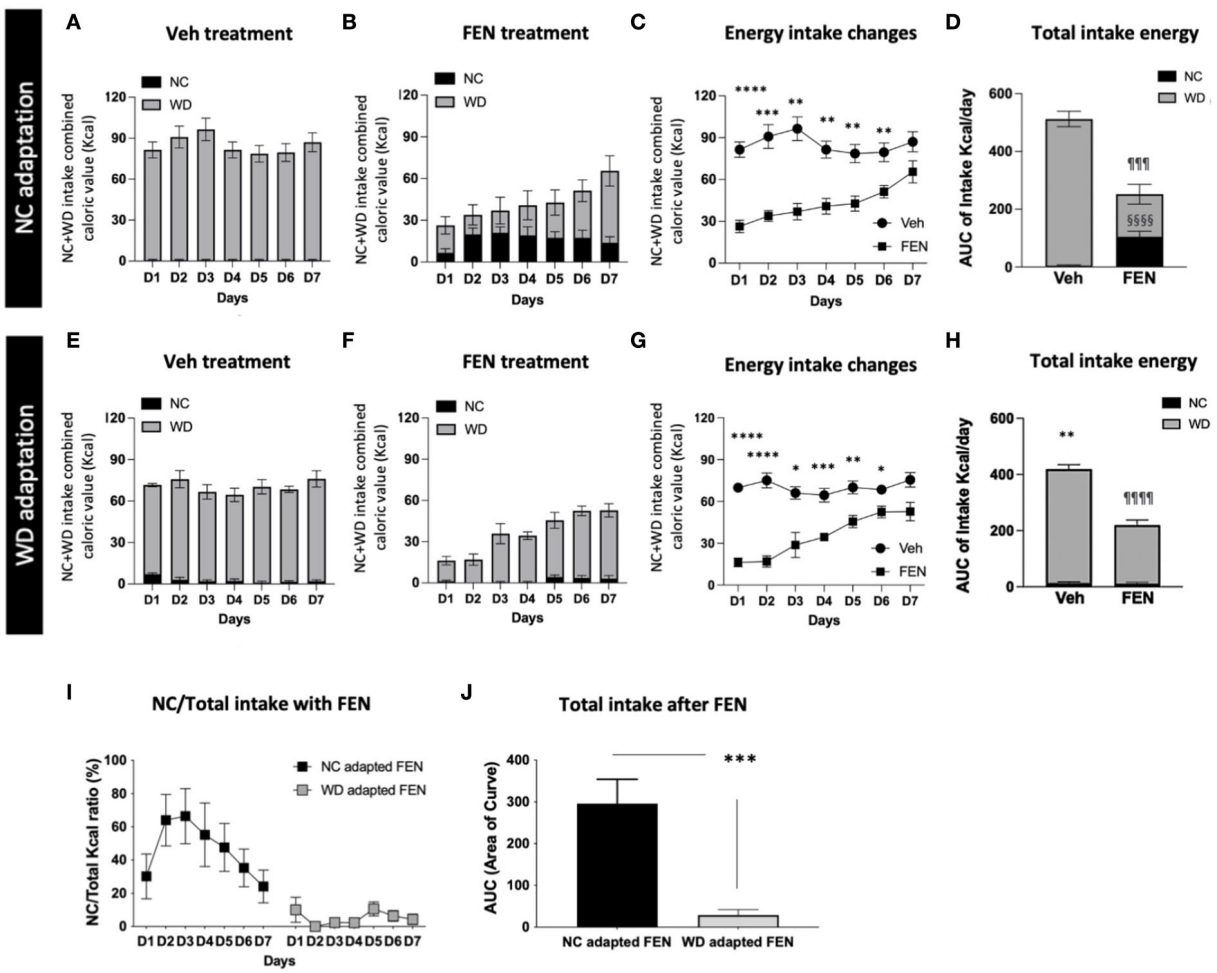
NC- or WD-food habituated animals (1 month) were divided into vehicle (SAL) and FEN groups, respectively. They were habituated to just one type of food for 1 month (NC or WD) and exposed to different types of food (WD or NC). Then they had free access to both foods, with a 2-day adaptation period before drug administration. Calorie intake of NC + WD food of **(A)** Veh-treated, and **(B)** FEN-treated animals of NC-adapted animals, **(C)** changes in energy intake of each group, and **(D)** a comparison of NC and WD intake among total calories. Calorie intake of NC + WD food of **(E)** Veh-treated and **(F)** FEN-treated animals of WD-adapted animals, **(G)** changes in energy intake of each group, and **(H)** a comparison of NC and WD intake among total calories. Total caloric intake of WD-adapted animals was significantly reduced throughout the administration period compared to NC-adapted animals. Error bars were expressed as mean ± SEM. ****p < 0.0001; ***p < 0.005; **p < 0.01; *p < 0.05 vs. Veh; ^§§§§^*p* < 0.0001 vs. Veh group for NC consumption, ^¶¶¶¶^*p* < 0.0001; ^¶¶¶^*p* < 0.005 vs. Veh group for WD consumption (Student's two-tailed *t*-test). **(I)** NC intake ratio (%) of NC- or WD-adapted animals after FEN administration and **(J)** a comparison of Akaike information criterion (AIC) values plotted by **(I)** a graph. NC-habituated and vehicle (SAL) injection group; NC-SAL, NC-habituated, and FEN injection group; NC-FEN, WD-habituated, and vehicle (SAL) injection group; WD-SAL, WD-habituated, and FEN injection group; and WD-FEN. ***p < 0.005 vs. NC adapted FEN (Student's two-tailed *t*-test). Error bars were expressed as mean ±SEM.

Because “Experiment I” suggested that increasing WD exposure diminishes FEN-induced suppression of WD, we extended “Experiment I” to access prolonged periods of WD consumption and FEN administration. Therefore, animals were habituated to NC or WD for 1 month, allowed to self-select between NC and WD for 2 days, and treated with saline or FEN for 7 days (Figure 1A). In Experiment I, animals habituated to NC for 1 month showed a significant shift to NC consumption, as demonstrated by a sharp increase in NC during the 2-day self-selection period, which was less evident in the 7-day self-selection period (Figures 3E,G). When given access to both diets, vehicle-treated NC-habituated animals (NC-SAL) consumed mostly the palatable WD food (Figure 4A). Both NC- and WD-habituated groups responded immediately to the anorexic effects of FEN on day 1 (Figures 4B,C,F,G; ^****^p < 0.0001; ^***^p < 0.005; ^**^p < 0.01; ^*^p < 0.05) and demonstrated the development of insensitivity to FEN over the 7-day reatment period (Figures 4B–D,F–H). As observed in Experiment I, NC-habituated animals treated with FEN showed a shift toward NC consumption compared to those in the vehicle-treated group (Figures 4A,B). These results can be observed in more detail in Figures 4C–E. The initial decrease in feed caloric intake observed in the FEN-administered group recovers to the level of the Veh group over time (Figure 4C). However, the FEN group showed a significant decrease in total caloric intake and, in particular, the NC food intake, which was not seen in the Veh group, showed a statistically very prominent result (^§§§§^*p* < 0.0001; Figure 4D). NC intake in the WD-FEN group was significantly lower when compared to the NC intake consumed by the NC-FEN group (^***^p < 0.001; Figures 4I,J). WD-adapted animals showed very different results from the food intake tendencies of NC-adapted animals after FEN administration. As seen, these animals showed no interest in NC food and only craved WD during FEN treatment (Figures 4E–H). Interestingly, in the results of the animals administered with Veh, the total caloric intake of WD-adapted animals was found to be significantly reduced throughout the administration period compared to NC-adapted animals (Figures 4A,D,E,H; ^**^p < 0.01). Further studies will be necessary to determine how chronic WD intake alters the homeostatic regulation of caloric intake. Although WD resulted in nearly all calories while animals had access to both diets, animals that had long-term exposure to WD did not demonstrate a shift toward NC consumption from D1 after FEN treatment (^¶¶¶¶^*p* < 0.0001; Figures 4F–H) even though there was still an immediate response in total caloric intake to FEN compared to vehicle-treated animals (Figures 4B–D,F–H).

### Experiment III: Observing the effects of different FEN concentrations on the food preference of NC-habituated animals

**Figure 5:**
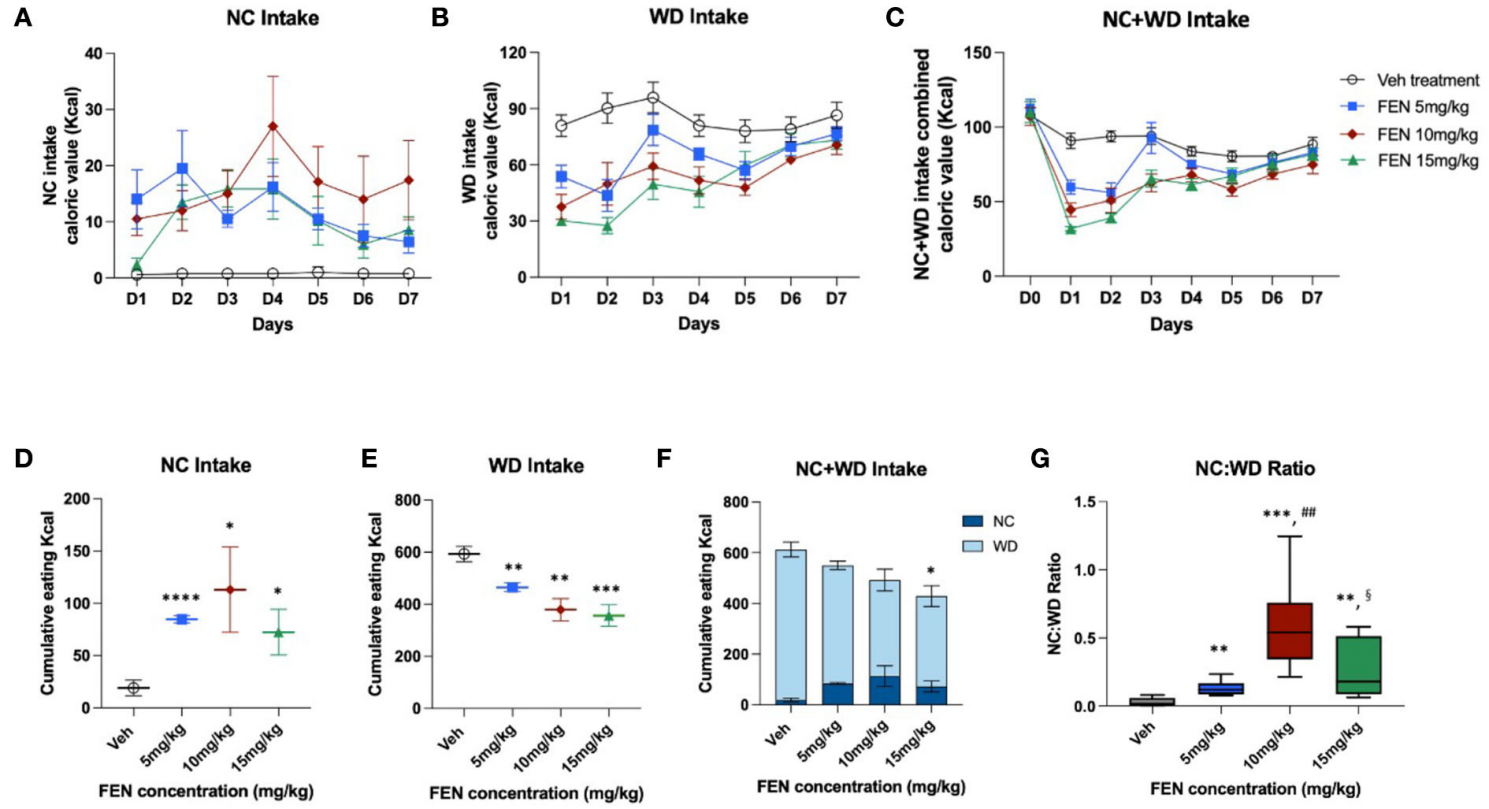
NC-habituated animals (1 month) were observed to see if different concentrations of FEN affected their eating behavior and preferences regarding the ingredients of different foods (NC and WD). It was administered for 7 days after a 2-day adaptation period. **(A)** NC calorie intake, **(B)** WD intake, **(C)** total calorie intake (NC + WD) under four dosing conditions, **(D)** cumulative NC calorie intake values, **(E)** WD calorie intake values, **(F)** total calorie intake values according to the concentrations of FEN, and **(G)** the ratio of NC and WD intake with increasing FEN concentration. ****p < 0.0001; ***p < 0.005; **p < 0.01; *p < 0.05 vs. Veh; ^##^*p* < 0.01 vs. FEN (5 mg/kg); ^§^*p* < 0.05 vs. FEN (10 mg/kg). Error bars were expressed as mean ± SEM (Student's two-tailed *t*-test).

This study examined whether the variation in FEN concentrations would be different after NC-habituated feeding behavior when animals had a choice between two calorically diverse diets. As shown in Figure 5, the overall eating trends in all FEN-treated groups were very similar after the 2-day adaptation period. According to the results, FEN administration with three different doses (5, 10, and 15 mg/kg) showed similar patterns of NC and WD consumption over 7 days (Figures 5A–C). FEN treatment dose-dependently decreased caloric intake on D1, which decreased with each subsequent day (Figures 5B,C, *p* < 0.01 vs. Veh treatment). Moreover, FEN-induced transition to NC was observed at all drug concentrations, although it did not appear to do so in a dose-dependent manner (Figure 5D, not significant between the administration groups). However, when both types of foods were consumed simultaneously, the total accumulated calories showed a statistical difference from the Veh-administered group only when FEN reached 15 mg/kg (Figure 5F). In addition, it was confirmed that when the concentration of FEN was increased, total calories consumed according to the concentration decreased (Figure 5F). We found that the NC:WD ratio increased after FEN administration compared to the Veh-treated group (Figure 5G). However, the deviation from the measured values was quite considerable at high concentrations of FEN (10 and 15 mg/kg). FEN (10 mg/kg) showed a significantly higher NC:WD ratio than FEN (15 mg/kg). Therefore, regardless of the FEN concentration, total caloric intake recovered to the same level as before FEN administration. Still, the NC:WD ratio seems to rise at a high concentration than that of FEN (5 mg/kg). However, it is noteworthy that the NC:WD ratio in FEN (15 mg/kg) decreases significantly compared to that in FEN (10 mg/kg).

## Discussion

A disadvantage of daily administration of FEN is that chronic treatment with the drug leads to pharmacological tolerance after a few days (Choi et al., 2002, 2006; Fernstrom and Choi, 2008). Many anti-obesity and antidepressant drugs share this pharmacological pattern (Li and Cheung, 2011). However, although many researchers and physicians have attempted to reveal the exact pharmacological mechanisms, their causes are not well understood (Adler and Stewart, 2009). Anti-obesity drugs can show special effects in some patients; however, sometimes they do not. Our data suggest that the long-term experience with food of different caloric values can affect the success of obese patients treated with anti-obesity drugs and subsequently lose its pharmacological effect during chronic treatment.

Previous studies have shown that long-term exposure to specific food causes different eating behaviors in humans (Epstein et al., 2009, 2011), which can affect the efficacy of anti-obesity drugs. Currently, a few studies have explored the effects of a short-term additional exposure to a high-fat and high-carbohydrate diet (WD) or long-term WD intake with repeated FEN administration (Figure 1). In our study, animals had access to NC or a WD for 2 or 7 days, and all groups preferred the consumption of WD over NC. As predicted, Experiment I with FEN significantly demonstrated that even short periods of WD access could dramatically influence the animals' food selection (Figures 3E,G), as the 2-day adaptation group (Figure 3B) showed a considerable shift toward NC consumption in response to FEN administration (Figure 3E). On the other hand, the 7-day adaptation group (Figure 3C) did not appear to alter the ratio of NC consumption after FEN treatment compared to the 2-day adaptation group (Figure 3G). This suggests that exposure to a calorically dense diet for 1 week alters their feeding preferences during the hypophagic phase of FEN administration. More extended periods (30 days) of high caloric intake of a palatable food with high-fat content did not alter the ratio of NC and WD intake after FEN administration from that observed after 7-day WD intake (Figure 4). In contrast, if an animal is habituated with NC and then exposed to a novel palatable food such as WD, it significantly loses their preferences for the edible food when treated with FEN (Figures 3, 4). Although we performed an animal experiment with the same design (Figures 3D,E, 4A,B), the numbers shown in the results may not be precisely the same (Choi et al., 2002, 2004, 2006, 2011). Although these results often occur in an independent set of experiments, what we need to focus on in these results is that when FEN is administered to NC-adapted animals, the animals are not only interested in WD but also repeatedly confirm their interest in NC intake. These results could be significant within a clinical setting when considering the effectiveness of anti-obesity drugs and long-rooted food preferences (Vabø and Hansen, 2014). Diets in the western food culture are primarily based on high-fat foods compared to eastern food cultures (Lupton, 2000; Lartey et al., 2018; de Mestral et al., 2019). In addition, individual differences bring an added level of complexity and variation to dieting patterns (Lupton, 2000; Lartey et al., 2018; de Mestral et al., 2019). Thus, our data suggest that anti-obesity drugs (e.g., FEN) can yield very different results based on the macronutrient content of the food to which patients are habituated (Choi et al., 2011; Sherafat-Kazemzadeh et al., 2013).

Although these results were obtained with FEN, a serotonin-specific reuptake inhibitor (SSRI), other SSRIs or appetite-suppressing treatments may have similar outcomes. These results broadly suggest that the general macronutrient composition of a patient's diet should be considered when evaluating the effectiveness of anti-obesity drugs.

When the concentration of FEN treatment was increased, WD intake was dose-dependently suppressed and persisted within the first 5 days of treatment, supporting previous studies (Choi et al., 2002; Fernstrom and Choi, 2008). However, escalating FEN treatment did not result in a dose-dependent relationship for concomitant switches involving NC intake (Figures 5A,D), suggesting that the transition to NC is not driven by macronutrient intake ratios *per se*. It was found that, as the concentration of FEN increased, cumulative calorie intake decreased (Figure 5F), and the intake rate of WD decreased (Figures 5E,F). However, the pattern of caloric intake with repeated doses of FEN was similar regardless of the dose (Figure 5C).

Fenfluramine is no longer prescribed in the USA for weight loss; it was taken off from the market because of cardiac side effects. Nevertheless, not only has FEN been used as a representative SSRI agent for the past 30 years, but information on various mechanisms of appetite suppression is abundant, suggesting that the effects of FEN are similar to those of SSRI agents for appetite suppression. Previously, Hurley et al. (2016, 2019) reported that PACAP could attenuate palatability-induced feeding by reducing the perceived hedonic value of palatable foods. Similarly, FEN suppresses hedonic features of the diet, whereas the return to NC may be homeostatic caloric regulation (Bojanowska and Ciosek, 2016). This current study goes beyond the simple conclusion that drugs may specifically act on specific areas of the brain to indicate differences in the choice of palatable foods. This study found that the dietary habits they had before applying FEN, as well as the experience and duration of any macronutrient, could act as another significant variable in determining its pharmacological effects.

Therefore, daily administration of appetite suppressants such as FEN is of concern for a variety of pharmacological benefits for individuals with specific macronutrient ratios and eating histories.

## Conclusion

In summary, our study corroborates that there is a decline in the pharmacological effects of FEN after chronic treatment; however, its anti-appetite effects may be differentially affected by the type of habituated macronutrient history. Thus, the habituation of preference for specific nutrition ratios found in obese patients is crucial to anticipate the pharmacological success of anti-obesity drugs before drug resistance develops.

## Data availability statement

The original contributions presented in the study are included in the article/supplementary material, further inquiries can be directed to the corresponding authors.

## Ethics statement

All procedures were performed in accordance with the guidelines and approval of the Marquette University Institutional Animal Care and Use Committee (IACUC).

## Author contributions

SY was responsible for designing the experimental protocols, conducting the search, screening potentially eligible studies, extracting and analyzing data, manuscript draft writing, and interpreting results. SC was responsible for designing the experimental protocols, extracting and analyzing data, and completing manuscript writing. All authors contributed to the article and approved the submitted version.

## Funding

This work was supported by the NIH NIDDK 074734 to SC; the National Research Foundation of Korea grant funded by the Korean Government (NRF-2018R1D1A3B07047960) and Soonchunhyang University Research Fund to SY.

## Conflict of interest

The authors declare that the research was conducted in the absence of any commercial or financial relationships that could be construed as a potential conflict of interest.

## Publisher's note

All claims expressed in this article are solely those of the authors and do not necessarily represent those of their affiliated organizations, or those of the publisher, the editors and the reviewers. Any product that may be evaluated in this article, or claim that may be made by its manufacturer, is not guaranteed or endorsed by the publisher.
